# Benchmarking and efficiency assessment in intensive care units: a systematic review protocol

**DOI:** 10.62675/2965-2774.20260280

**Published:** 2026-01-28

**Authors:** Luís Filipe Azevedo de Oliveira, Fernando Luiz Cyrino Oliveira, Leonardo dos Santos Lourenço Bastos, Giulliana Martines Moralez, Jorge Ibrain Figueira Salluh, Igor Tona Peres

**Affiliations:** 1 Pontifícia Universidade Católica do Rio de Janeiro Department of Industrial Engineering Rio de Janeiro RJ Brazil Department of Industrial Engineering, Pontifícia Universidade Católica do Rio de Janeiro - Rio de Janeiro (RJ), Brazil.; 2 Instituto D’Or de Pesquisa e Ensino Rio de Janeiro RJ Brazil Instituto D’Or de Pesquisa e Ensino - Rio de Janeiro (RJ), Brazil.

**Keywords:** Intensive care unit, Efficiency, Benchmarking, Systematic review protocol

## Abstract

**Objective:**

To identify research gaps and propose strategies for enhancing the quality and efficiency of critical care services globally.

**Methods:**

We will explore and analyze metrics, models, and methodologies for efficiency assessment and benchmarking in intensive care units, including both static and longitudinal approaches. For so, we will comprehensively search three electronic databases (Scopus, Web of Science, and Embase) using predefined keywords combined with Boolean operators. The search will target peer-reviewed studies without time frame restrictions. Duplicates will be removed, and two reviewers will independently assess the eligibility of articles based on predefined inclusion and exclusion criteria. Relevant data will be extracted and organized thematically using a standardized form aligned with PRISMA guidelines. The extracted data will include a study of characteristics, methodologies, and outcomes, enabling structured mapping and synthesis of existing evidence. This review does not require ethical approval.

**Results:**

The findings will be disseminated through open-access journal publications and national and international conferences presentations. The results will also be shared with key stakeholders, including healthcare professionals, policymakers, and researchers, to foster discussions on improving intensive care unit performance.

**Conclusion:**

This review will synthesize available evidence on ICU efficiency assessment, contributing to standardization efforts and guiding future research priorities aimed at strengthening critical care management and benchmarking practices worldwide.

## INTRODUCTION

Performance assessment and benchmarking in intensive care units (ICUs) are fundamental to improving the quality and effectiveness of critical care. These evaluations are usually conducted over time or by comparing ICUs across sites or regions.^(1,2)^ Critical care databases, such as ICU registries,^(3–5)^ have become a key enabler of ICU evaluation, supporting data-driven management and benchmarking processes.^(6,7)^ This approach provides a comprehensive understanding of disparities in care and fosters evidence-based improvements in care delivery.^(8,9)^

Regarding the results and indicators used to compare critical care units, several measures have been proposed.^(10–13)^ Mortality is widely recognized as a Key Performance Indicator (KPI) and a central quality measure in ICUs, as it directly correlates with other patient outcomes and reflects the effectiveness of medical interventions and the overall quality of care provided.^(14)^ Length of stay (LOS), in both hospital and ICU, is another commonly used KPI that reflects not only cost and efficiency, but also the efficacy of care processes. While efficacy is typically assessed at specific points in time as discrete outcomes, efficiency reflects a longitudinal perspective, evaluating resource use and outcomes across the entire care episode.^(12,15,16)^ However, both mortality and LOS are influenced not only by structural factors, patient transfers, case-mix, and severity of illness, but also by pre-ICU care, admission and discharge criteria, end-of-life care practices, and local patterns of ICU utilization.^(17,18)^

To enable fair comparisons across ICUs, risk adjustment is essential. Severity scoring systems such as the Acute Physiology and Chronic Health Evaluation (APACHE), the Simplified Acute Physiology Score (SAPS), and the Mortality Probability Models (MPM) are widely used to estimate mortality risk based on physiological parameters, comorbidities, and admission characteristics.^(19)^ In addition, locally developed models such as the Australia and New Zealand Risk of Death (ANZROD)^(20)^ and the Intensive Care National Audit and Research Centre (ICNARC) model^(21)^ in the United Kingdom play a central role in benchmarking within specific national contexts. These tools enable the calculation of risk-adjusted outcomes, ensuring that benchmarking reflects differences in care quality rather than variations in case-mix.^(7)^

Different methods have been considered for ICU outcome benchmarking. Previous studies compared performance using ranking tables^(22)^ or funnel plots^(23,24)^ for single metrics. A dual-metric approach, like the "efficiency matrix",^(25,26)^ combines risk-adjusted mortality and resource use. More recently,^(27,28)^ the average standardized efficiency rate (ASER) has been proposed as an alternative to integrate the standardized mortality ratio (SMR) and the standardized resource use (SRU) into a single parameter, offering a complementary approach to the efficiency matrix. Unsupervised learning methods (e.g., clustering) also show potential to identify ICUs with similar performance.^(22)^

Methods from business benchmarking, such as Data Envelopment Analysis (DEA) and Stochastic Frontier Analysis (SFA), address multiple inputs and outputs and provide individual targets for efficiency improvement.^(29,30)^ Data Envelopment Analysis and SFA models have been widely used in healthcare systems nationwide, but less in ICU benchmarking.^(31)^ Importantly, both DEA and other statistical models rely on risk-adjusted outcomes, especially for metrics like mortality and LOS.

The literature suggests that critical care efficiency should be evaluated using performance indicators, validated in clinical research and observational studies.^(5,16)^ However, challenges exist in measuring these indicators, including variability in definitions and methods, which compromise the replicability of assessments and international benchmarking.^(5,32)^ Benchmarking outcomes in critical care, supported by risk adjustment models and advanced techniques like machine learning, is essential for performance evaluation and continuous quality improvement in ICUs, providing critical guidelines for clinical practice and healthcare management.^(24,33)^ Moreover, systematic benchmarking and continuous outcome monitoring enable the identification of best practices, which, when incorporated into care processes, can strengthen organizational resilience. This contributes to more effective crisis management, enhances day-to-day performance, and supports an adaptive response to diverse challenges, ensuring consistent delivery of high-quality care.^(34)^

Given the critical role of efficiency assessment in improving critical care, consolidating existing knowledge on the best metrics, models, and practices for evaluating ICU performance is essential. Despite available approaches, the current state of the art regarding methods for describing ICU efficiency, their advantages and limitations, and appropriate circumstances remains unclear. This consolidation must encompass both qualitative and quantitative dimensions, addressing identified methodological and contextual challenges. In this context, the present study proposes a protocol to conduct a systematic review to explore, identify, and analyze metrics, models, organizational characteristics, and the association between care processes and ICU efficiency, along with the influence of adherence to these processes on efficiency outcomes. Our main objective is to identify research gaps and propose strategies for enhancing the quality and efficiency of critical care services globally. The specific objectives of this review are:

– To describe the indicators used to study efficiency in ICU settings.
– To identify the most suitable models for evaluating ICU efficiency, considering static and longitudinal approaches.
– To examine the organizational variables (structure and process) associated with superior ICU performance.
– To analyze what processes of care were related to efficiency and how adherence to these care processes relates to ICU efficiency.

## METHODS

This protocol was structured according to the Preferred Reporting Items for Systematic reviews and Meta-Analyses for Protocols (PRISMA-P)^(35)^ statement. The systematic review will be conducted according to the guidelines of the PRISMA.^(36–38)^ The protocol was registered in the international database of systematic reviews, PROSPERO (registration number CRD42025630494), on 22 Dec 2024, following the initial literature search, and subsequently updated on 20 Jan 2025.

### Context and concept

This systematic review aims to evaluate efficiency and benchmarking practices in ICUs. The following definitions and concepts will guide the scope of the review:

1. Intensive Care Units are specialized hospital units designed to provide comprehensive, continuous care to critically ill patients.^(39)^ These units typically employ advanced monitoring technologies, highly trained personnel, and life-support interventions to manage complex medical conditions.^(40)^
2. Efficiency in ICUs is the optimal utilization of available resources to achieve desired health outcomes while balancing quality and cost-effectiveness in patient care.^(41)^ It reflects how well inputs (e.g., staff, equipment, beds) are transformed into outputs and outcomes (e.g., survival, functional recovery, patient satisfaction) in a technically sound and sustainable manner.^(29)^
3. Benchmarking in ICUs involves comparing performance metrics across units, hospitals, or regions to identify the best practices and areas for improvement. These evaluations can include static analyses (assessing performance at a single point in time) and longitudinal analyses (tracking performance over time).^(24)^
4. Quality is an overarching concept that encompasses not only outcome measures (e.g., mortality and functional recovery) but also process measures (e.g., adherence to evidence-based protocols, which are directly related to efficacy) and the dimension of resource utilization (related to efficiency). This approach is consistent with Donabedian's^(42)^ structure-process-outcome model and with the principles of value-based healthcare, which emphasize achieving the best health outcomes while optimizing resource use. By explicitly framing quality in this multidimensional manner, we ensure conceptual clarity and alignment with contemporary performance assessment frameworks in intensive care.^(43)^
5. ICU performance indicators: the review emphasizes commonly used indicators, including mortality rates, LOS (both in ICUs and hospitals), adherence to evidence-based protocols, resource utilization, and other patient outcomes.^(44)^ These indicators are crucial for assessing the quality, efficiency, and equity of critical care delivery.^(24)^
6. Performance assessment methodologies: this study explores various approaches for evaluating efficiency and benchmarking in ICUs. These include quantitative techniques such as DEA, econometric models, machine learning, logistic regression, and other advanced statistical methods, as well as qualitative analyses (e.g., Patient-Reported Outcome Measures [PROMs] and Patient-Reported Experience Measures [PREMs]) that incorporate contextual and organizational factors. Integrating these approaches enables a more comprehensive and nuanced understanding of ICU efficiency.^(33,41,45)^

This conceptual framework provides the foundation for systematically exploring the metrics, models, methodologies, and variables that impact ICU efficiency. It fosters a deeper understanding of benchmarking practices and their implications for healthcare systems worldwide.

### Search strategy

The search strategy was developed to systematically identify studies on efficiency assessment and benchmarking in ICUs. The query combined terms related to ICU settings (e.g., "intensive care*", "icu*", "critical care*", "critical ill*", "critically ill*"), evaluation processes (e.g., "compar*", "evaluat*", "assess*"), performance concepts (e.g., "efficienc*", "benchmark*", "resilien*"), and formal analytical approaches (e.g., "data envelopment analy*", "DEA", "machine learning", "regression", "model*", "SMR", "SRU"). Truncation symbols (*) were used to capture variations in word endings and maximize retrieval.

The query will be restricted to the title, abstract, and keyword fields to enhance precision and ensure that the selected articles addressed the core concepts of the review. Complete search strategies, including syntax adaptations for Scopus, Web of Science, and Embase, are presented in table 1S (Supplementary Material).

### Study selection

The study selection process involved four main steps: (i) definition of inclusion and exclusion criteria; (ii) title and abstract screening; (iii) full-text eligibility assessment; and (iv) inclusion of additional studies through backward and forward citation tracking.

Studies will be eligible for inclusion if they assessed ICU efficiency using formal, structured, and replicable methodologies (quantitative or qualitative approaches applying analytical models, efficiency analysis techniques, or robust conceptual frameworks) or if they performed benchmarking of ICUs based on systematic comparisons with explicit criteria. Eligible studies must report metrics, models, or evaluations directly related to ICU performance (operational, clinical, managerial, or organizational). Studies involving healthcare professionals, managers, patients, or stakeholders directly engaged in ICU care or management will also be included, as their participation may influence how efficiency is structured, perceived, and evaluated in different healthcare settings. Additionally, studies analyzing variations in ICU performance at regional, national, or international levels will be included.

Exclusion criteria will include studies conducted outside ICU settings (e.g., intermediate care units, emergency or urgent care services); those exclusively focused on pediatric or neonatal populations; studies lacking sufficient methodological detail (i.e., without a clear description of metrics, models, variables, or analytical procedures); and those addressing only clinical or pharmacological outcomes unrelated to efficiency or benchmarking. Also excluded will be studies based solely on predictive models for individual patient outcomes (e.g., mortality risk, sepsis risk, LOS) unless such models are explicitly applied to compare ICU performance. Studies proposing only recalibration of traditional prognostic models without direct benchmarking application or performing benchmarking exclusively between prognostic models without comparison of ICU performance will also be excluded. Furthermore, non-peer-reviewed publications (e.g., abstracts, theses, white papers, opinion articles, narrative reviews), studies with insufficient information on efficiency or benchmarking processes, and those not published in English (unless a complete translation is available) will be excluded to ensure methodological consistency and accuracy in data extraction and interpretation.

Two independent reviewers will apply these criteria during the screening stages. Disagreements will be resolved through discussion or consultation with a third reviewer.

### Data extraction

The screening process will be carried out using the Rayyan software (http://rayyan.qcri.org), a free tool that assists with screening titles and abstracts.^(46)^ Additionally, the ASReview software will implement active learning during the triage phase. This approach leverages machine learning algorithms to prioritize studies for manual review, thereby optimizing the screening process.^(47)^ Two independent reviewers will conduct the initial screening, evaluating the titles and abstracts of the retrieved records based on predefined inclusion and exclusion criteria. Selected articles will be thoroughly evaluated for eligibility.

### Risk of bias

We will assess the risk of bias of included studies at the study level using an adapted framework informed by the Prediction model Risk of Bias Assessment Tool (PROBAST)^(48)^ and the Transparent Reporting of a multivariable prediction model for Individual Prognosis Or Diagnosis (TRIPOD)^(49)^ guideline, complemented by methodological guidance for observational health services research. The assessment will focus on six domains relevant to ICU efficiency and benchmarking: (i) representativeness of the study population and potential selection bias; (ii) clarity and consistency in the definition of input variables and predictors; (iii) adequacy of outcome measures (e.g., mortality, LOS, resource utilization); (iv) appropriateness of analytical approaches and risk adjustment procedures; (v) completeness and reliability of data sources; and (vi) transparency of reporting and potential selective outcome reporting. Each domain will be rated as low, high, or unclear risk of bias. Studies will be considered to have a high overall risk of bias if any domain is rated as high risk. Two reviewers will independently complete the assessment, with disagreements resolved through discussion or consultation with a third reviewer.

### Data mapping

Upon completing data extraction, the collected literature will be thematically organized. This process will serve as the foundation for data mapping, during which key concepts related to ICU efficiency and benchmarking will be identified and structured into a conceptual framework to guide the comprehension of available evidence. This will enable the synthesis of existing findings by comparative analysis and grouping of approaches, highlighting patterns and trends in the literature regarding these assessments’ practices and outcomes. An electronic spreadsheet developed in accordance with the PRISMA guidelines will be used to document the characteristics of included studies and relevant key information. This includes not only general descriptors such as authors, year of publication, country of origin, study design, objectives, sample size, and comparator groups, but also domain-specific variables such as:

– Efficiency models and analytical frameworks (e.g., DEA, SFA, regression-based models, machine learning techniques);
– Input variables (e.g., number of ICU beds, staff-to-patient ratio, availability of medical equipment);
– Output and outcome measures, distinguishing how each study defines efficiency (the technical relationship between inputs and outputs, e.g., SRU, LOS, bed occupancy rates, cost-effectiveness) and/or quality (the nature of the outputs, e.g., SMR, functional outcomes, patient satisfaction, readmission rates), while explicitly avoiding the conflation of resource utilization with outcome measurement;
– Contextual factors (e.g., hospital type, ICU specialization, geographic region, funding model).

The primary findings will be presented in tabular and narrative formats to characterize ICU efficiency and benchmarking practices. The synthesis process will aim to summarize existing research and identify gaps in the literature, particularly in areas requiring further investigation to expand understanding and guide future research and practices in critical care performance assessment.

To ensure these findings’ practical relevance and applicability, this review protocol will incorporate key methodological considerations, including adapting to the specific health context of ICUs, employing case studies, iteratively refining practices, and integrating expert feedback. This approach aims to provide insights for policymakers, healthcare managers, and researchers, ensuring that benchmarking and efficiency evaluation strategies are tailored to the diverse operational contexts of ICUs.

## DISCUSSION

This study will contribute to a comprehensive understanding of the methodologies and practices used in ICU efficiency assessment and benchmarking. This comprehensive understanding, derived from the identification and analysis of diverse metrics, models, and variables, is crucial for ensuring high-quality care and optimal resource allocation in complex hospital environments. Previous research, such as that conducted by Soares et al.^(17,50)^ and Zampieri et al.,^(51–53)^ demonstrates that organizational characteristics, such as the implementation of protocols and the holding of multidisciplinary meetings, are directly associated with reduced mortality and more efficient resource utilization. Furthermore, scoring systems such as the SAPS, APACHE, and SMS-ICU have been widely adopted for assessing patient severity and predicting clinical outcomes, underscoring the importance of integrating clinical and operational data to enhance hospital management.

Contemporary approaches, including machine learning and hybrid methods that integrate DEA with machine learning, are emerging as significant alternatives for ICU performance evaluation. Advanced statistical models, such as linear regressions, mixed models, and machine learning-based methods, such as neural networks and random forests, offer substantial potential for accurately measuring ICU performance,^(12,13,54)^ such as those based on the ICU LOS, which serves as a proxy for allocative efficiency and contributes to benchmarking analysis. Mortality prediction models commonly used in ICUs also play a critical role in risk stratification and in the quality assessment of services provided.^(7)^ Additionally, hybrid approaches integrating DEA with neural networks have proven effective for predicting performance and identifying resilience patterns in hospitals and ICUs.^(54,55)^ Nevertheless, despite their predictive power, advanced machine learning models raise concerns regarding interpretability and clinical trust.^(56)^ Balancing predictive performance with explainability remains a key challenge for their implementation in ICU practice.^(7)^

Beyond optimizing efficiency and quality, this review is crucial for understanding how evaluation methodologies can incorporate organizational resilience, an essential attribute for ICUs to adapt to unexpected demands and sustain quality care. According to Salluh et al.,^(34)^ the resilient ICU can adapt to crises or sudden changes in case mix, staffing, or patient volume with minimal impact on clinical outcomes, and can learn from experience to sustain and improve practices over time. Salluh et al.^(34)^ add that resilience can be assessed using the "4S" framework (staff, stuff, space, and systems), complemented by a fifth "S" (science), which reflects the unit's capacity for continuous learning and adaptation.

While this study anticipates significant contributions, it acknowledges potential limitations, including the heterogeneity of available models and metrics and variations in the quality of the included studies. The absence of relevant literature in languages other than English may also limit the representativeness of the findings. This heterogeneity is further compounded by significant variability in the effectiveness of intensive care across regions and contexts, influenced by factors such as critically limited resources, differences in care quality and intervention types, and infrastructure, staff training, and access to advanced technologies.^(57,58)^

The findings of this systematic review will be disseminated through publications in academic journals and presentations at national and international conferences. Moreover, the results will be shared with policymakers, health managers, and researchers to foster discussions on how the findings can inform clinical practice, public policy development, and resource allocation. This work is also expected to serve as a foundation for developing new research questions, contributing to methodological and strategic advances in ICU efficiency evaluation.

## Data Availability

The contents are already available.
