# Supplementary material for: Benchmarking and efficiency assessment in intensive care units: a systematic review protocol
Source: Crit Care Sci. 2026 Jan 28;38:e20260280. doi: 10.62675/2965-2774.20260280 (PMC12977217; doi:10.62675/2965-2774.20260280)
Supplement: Supplementary Material [file 2965-2774-ccsci-38-e20260280-suppl1.pdf]

Critical Care Science
https://doi.org/10.62675/2965-2774.20260280

SUPPLEMENTARY MATERIAL

Benchmarking and efficiency assessment in intensive care units: a systematic review protocol

Luís Filipe Azevedo de Oliveira[1], Fernando Luiz Cyrino Oliveira[1], Leonardo dos Santos Lourenço Bastos[1], Giulliana Martines Moralez[2], Jorge Ibrain Figueira Salluh[2], Igor Tona Peres[1]

Table 1S - Search strategy

Data base	Search query
Scopus	TITLE-ABS-KEY ((“intensive care*” OR “icu*” OR “critical care*” OR “critical ill*” OR “critically ill*”) AND (“compar*” OR “evaluat*” OR “assess*”) AND (“efficienc*” OR “performance*” OR “benchmark*” OR “resilien*”) AND (“data envelopment analy*” OR “dea” OR “machine learning” OR “regression” OR “model*” OR “SMR” OR “SRU”) AND NOT (“neo*natal*” OR “child*” OR “kid*” OR “pediatric*” OR “infant*” OR “muscle*” OR “image classification unit” OR “animal*” OR “rat” OR “cadaver*” OR “home” OR “behav**” OR “spiritual*” OR “moral*” OR “cognitive*” OR “extracorporeal*” OR “pharmac*” OR “bact*” OR “ultrasound*” OR “chatGPT” OR “transfusion*” OR “embolism*” OR “facial” OR “pain” OR “sleep” OR “gastr*”))
Embase	(‘intensive care*’:ti,ab,kw OR ‘icu*’:ti,ab,kw OR ‘critical care*’:ti,ab,kw OR ‘critical ill*’:ti,ab,kw OR ‘critically ill*’:ti,ab,kw) AND (‘compar*’:ti,ab,kw OR ‘evaluat*’:ti,ab,kw OR ‘assess*’:ti,ab,kw) AND (‘efficienc*’:ti,ab,kw OR ‘performance*’:ti,ab,kw OR ‘benchmark*’:ti,ab,kw OR ‘resilien*’:ti,ab,kw) AND (‘data envelopment analy*’:ti,ab,kw OR ‘dea’:ti,ab,kw OR ‘machine learning’:ti,ab,kw OR ‘regression or model*’:ti,ab,kw OR ‘smr’ OR ‘sru’:ti,ab,kw) NOT (‘neo*natal*’:ti,ab,kw OR ‘child*’:ti,ab,kw OR ‘kid*’:ti,ab,kw OR ‘pediatric*’:ti,ab,kw OR ‘infant*’:ti,ab,kw OR ‘muscle*’:ti,ab,kw OR ‘image classification unit’:ti,ab,kw OR ‘animal*’:ti,ab,kw OR ‘rat or cadaver*’:ti,ab,kw OR ‘home or behav*’:ti,ab,kw OR ‘spiritual*’:ti,ab,kw OR ‘moral*’:ti,ab,kw OR ‘cognitive*’:ti,ab,kw OR ‘extracorporeal*’:ti,ab,kw OR ‘pharmac*’:ti,ab,kw OR ‘bact*’:ti,ab,kw OR ‘ultrasound*’:ti,ab,kw OR ‘chatgpt’:ti,ab,kw OR ‘transfusion*’:ti,ab,kw OR ‘embolism*’:ti,ab,kw OR ‘facial’:ti,ab,kw OR ‘pain’:ti,ab,kw OR ‘sleep’:ti,ab,kw OR ‘gastr*’:ti,ab,kw)
Web of Science	(TI=(“intensive care*” OR “icu*” OR “critical care*” OR “critical ill*” OR “critically ill*”)<br>OR AB=(“intensive care*” OR “icu*” OR “critical care*” OR “critical ill*” OR “critically ill*”)<br>OR AK=(“intensive care*” OR “icu*” OR “critical care*” OR “critical ill*” OR “critically ill*”))<br>AND<br>(TI=(“compar*” OR “evaluat*” OR “assess*”)<br>OR AB=(“compar*” OR “evaluat*” OR “assess*”)<br>OR AK=(“compar*” OR “evaluat*” OR “assess*”))<br>AND<br>(TI=(“efficienc*” OR “performance*” OR “benchmark*” OR “resilien*”)<br>OR AB=(“efficienc*” OR “performance*” OR “benchmark*” OR “resilien*”)<br>OR AK=(“efficienc*” OR “performance*” OR “benchmark*” OR “resilien*”))<br>AND<br>(TI=(“data envelopment analy*” OR “dea” OR “machine learning” OR “regression” OR “model*” OR “SMR” OR “SRU”)<br>OR AB=(“data envelopment analy*” OR “dea” OR “machine learning” OR “regression” OR “model*” OR “SMR” OR “SRU”)<br>OR AK=(“data envelopment analy*” OR “dea” OR “machine learning” OR “regression” OR “model*” OR “SMR” OR “SRU”))<br>NOT<br>(TI=(“neo*natal*” OR “child*” OR “kid*” OR “pediatric*” OR “infant*” OR “muscle*” OR “image classification unit”<br>OR “animal*” OR “rat” OR “cadaver*” OR “home” OR “behav**” OR “spiritual*” OR “moral*” OR “cognitive*”<br>OR “extracorporeal*” OR “pharmac*” OR “bact*” OR “ultrasound*” OR “chatGPT” OR “transfusion*”<br>OR “embolism*” OR “facial” OR “pain” OR “sleep” OR “gastr*”)<br>OR AB=(“neo*natal*” OR “child*” OR “kid*” OR “pediatric*” OR “infant*” OR “muscle*” OR “image classification unit”<br>OR “animal*” OR “rat” OR “cadaver*” OR “home” OR “behav**” OR “spiritual*” OR “moral*” OR “cognitive*”<br>OR “extracorporeal*” OR “pharmac*” OR “bact*” OR “ultrasound*” OR “chatGPT” OR “transfusion*”<br>OR “embolism*” OR “facial” OR “pain” OR “sleep” OR “gastr*”)<br>OR AK=(“neo*natal*” OR “child*” OR “kid*” OR “pediatric*” OR “infant*” OR “muscle*” OR “image classification unit”<br>OR “animal*” OR “rat” OR “cadaver*” OR “home” OR “behav**” OR “spiritual*” OR “moral*” OR “cognitive*”<br>OR “extracorporeal*” OR “pharmac*” OR “bact*” OR “ultrasound*” OR “chatGPT” OR “transfusion*”<br>OR “embolism*” OR “facial” OR “pain” OR “sleep” OR “gastr*”))

This is an open access article under the CC BY license (https://creativecommons.org/licenses/by/4.0/).
